# Comparative Study of Polysaccharide-Based Hydrogels: Rheological and Texture Properties and Ibuprofen Release

**DOI:** 10.3390/gels8030168

**Published:** 2022-03-07

**Authors:** Veronika Mikušová, Jarmila Ferková, Dominika Žigrayová, Daniel Krchňák, Peter Mikuš

**Affiliations:** 1 Department of Galenic Pharmacy, Faculty of Pharmacy, Comenius University Bratislava, Odbojárov 10, 83232 Bratislava, Slovakia; mikusova@fpharm.uniba.sk (V.M.); jarmila.ferkova@uniba.sk (J.F.); zigrayova1@uniba.sk (D.Ž.); krchnak6@uniba.sk (D.K.); 2 Department of Pharmaceutical Analysis and Nuclear Pharmacy, Faculty of Pharmacy, Comenius University Bratislava, Odbojárov 10, 83232 Bratislava, Slovakia; 3 Toxicological and Antidoping Center, Faculty of Pharmacy, Comenius University Bratislava, Odbojárov 10, 83232 Bratislava, Slovakia

**Keywords:** hydrogels, ibuprofen, rheologic and texture properties, drug release kinetics, in vitro diffusion study, polysaccharide polymers, chitosan

## Abstract

Polysaccharides are attractive gelling agents in pharmacy due to their safety, biocompatibility, biodegradability, relatively easy way of preparation, and low price. Due to their variable physical-chemical properties, polysaccharides have potentialities to be used for designing new drug delivery systems for controlled drug release. In this comparative study, rheological and texture properties as well as the in vitro release of model drug ibuprofen (IBU) with 11 polysaccharide-based hydrogels were investigated. The in vitro release of IBU significantly differed between (i) neutral (hydroxy/alkylcelluloses), (ii) anionic (carboxyalkylcellulose and its sodium salt, tragacanth, carrageenan, xanthan gum), and (iii) cationic (chitosans) hydrogels due to different contribution of provided interactions and viscosity within the hydrogel groups. The drug release kinetics of each hydrogel system was evaluated for five kinetic models. Several combinations of cationic hydrogels with neutral or anionic ones were performed to illustrate possibilities of providing modified IBU release profiles. In this context, chitosan was presented as an effective modifier of diffusion profiles for negatively charged drugs formulated into combined polymeric systems, providing their prolonged release. The most appropriate hydrogel for the topical application (i.e., providing favorable rheological and texture properties along with the highest drug release) was selected from a studied series of polysaccharide-based hydrogels.

## 1. Introduction

The development of pharmaceutical formulations involves the use of various excipients in addition to the active ingredient. In drug delivery, the excipient plays an important role in the drug release process, especially in modified release formulations. The drug release rate from the dosage form is controlled by the type and concentration of the excipients used in these formulations. Among various excipients, biopolymers are an attractive choice due to their low toxicity and immunogenicity, stability, biocompatibility, and biodegradability [1,2]. Some of the polysaccharide biopolymers frequently used as excipients in pharmaceutics are derived from plants (i.e., starch, cellulose, pectin, guar, tragacanth, and Arabic gum), algae (i.e., alginates, galactans, and carrageenans), animals (i.e., chitin, chitosan, glycosaminoglycans, hyaluronic acid), and microorganisms (i.e., dextran, bacterial cellulose, gellan gum, xanthan gum) [3]. Thus, their different monosaccharide composition, linkage types and patterns, the chain shape or length, and the molecular weight are responsible for their different physical-chemical properties, such as provided interactions (Van der Waals, H–bonds, Coulomb, steric, etc.), solubility, viscosity, gelling potential, and/ or surface and interfacial properties [4].

Natural polysaccharides are frequently used as base materials in hydrogels. Hydrogels are formed by crosslinked polymeric networks. They have a high affinity for water due to the presence of a great number of hydrophilic groups and are prevented from dissolving due to their crosslinked chemical or physical bonds among the polymer chains, resulting in enormous swelling with high water-holding capacity [5,6].

Cellulose is a natural polysaccharide consisting of a linear chain of β-(1→4) linked D-glucose units. Semi-synthetic derivatives of cellulose, namely, cellulose ethers (high molecular weight compounds produced by replacing the hydrogen atoms of hydroxyl groups in the anhydroglucose units of cellulose with alkyl or substituted alkyl groups, e.g., methylcellulose (MC), ethylcellulose (EC), hydroxyethylcellulose (HEC), hydroxypropyl cellulose (HPC), hydroxypropylmethylcellulose (HPMC), carboxymethyl cellulose (CMC), and sodium carboxymethyl cellulose (NaCMC), play important roles in different types of pharmaceuticals such as extended and delayed-release coated dosage forms, extended and controlled release matrices, osmotic drug delivery systems, bioadhesives and mucoadhesives, compression tablets as compressibility enhancers, liquid dosage forms as thickening agents, and stabilizers, granules, and tablets as binders, semisolid preparations as gelling agents, and many other applications [7,8]. Very recently, various combinations of cellulose ethers, namely, MC plus a water-soluble chitosan oligomer (CS-O) [9], NaCMC plus HPMC [10], hydroxyethylcellulose plus gellan gum [11], CMC plus polyethylene glycol (PEG) [12], were developed to improve the properties of resulting hydrogels and formulations of various model compounds and drugs (adenosine, l-ascorbic acid, methylene blue, tetracycline, phenylephrine, tropicamide, ketoconazole). In these ways, mechanical and self-healing properties, and drug release of the hydrogels were effectively improved.

Carrageenan (CRG) is a naturally occurring sulfated polysaccharide extracted from red algae by hot alkali separation, and consists of galactose and 3,6-anhydrogalactose linked by α-(1→3) and β-(1→4) glycosidic bonds [13]. It is currently a promising candidate in tissue engineering and regenerative medicine as it resembles native glycosaminoglycans [14]. Recently, Mahdavinia et al. [15] developed and evaluated ciprofloxacin-loaded hydrogel nanocomposites for sustained release of ciprofloxacin using chitosan/hydroxyapatite/κ-carrageenan complexes.

Tragacanth gum (TRG), a naturally occurring polysaccharide, is the exudate from Astragalus gummifer bark. Tragacanth is mainly a complex mixture of branched acidic hetero-polysaccharides containing d-galacturonic acid. It contains a minor amount of tragacanthin (water-soluble) and bassorin (arabinogalactan), which is swellable but not water-soluble. Tragacanth has been widely used in cosmetics, and pharmaceutical preparations as an emulsifier, thickener, and stabilizer for its moisture-retaining, binding, freezing, gelling, and adhesive properties [16]. Pathania et al. [17] used nanohydrogel of tragacanth gum for the controlled release of ampicillin. Tragacanth gum was grafted with itaconic acid, employing graft copolymerization in the presence of N,N1-methylene-bis-acrylamide (MBA) as cross-linker, and potassium persulphate as initiator.

Chitosan (CS) is a linear polysaccharide composed of randomly distributed β-(1→4)-linked D-glucosamine (deacetylated unit) and N-acetyl-D-glucosamine (acetylated unit). It is made by treating the chitin shells of shrimp and other crustaceans with an alkaline substance. Chitosan can be found in many dosage forms such as micro- or nanoparticles, sponges, films, or physical and chemical hydrogels [18]. Recently, Mahanta et al. [19] developed an injectable hydrogel of chemically modified chitosan for controlled drug delivery for model drugs tetracycline and doxorubicin. Chitosan was chemically modified through grafting of ester-diol-based polyurethane to transform into a hydrogel. In vitro drug release kinetics reveals that the graft copolymer releases the drug in a sustained manner as compared to the pure one.

Non-steroidal anti-inflammatory drugs (NSAIDs), such as ibuprofen (IBU), are amphiphilic substances capable of self-association in aqueous solutions and able to be sorbed onto polymers through hydrophobic and electrostatic bonds. The composition of the formulation has a significant impact on the delivery of IBU into the stratum corneum (SC), its subsequent transfer to the viable skin layers, and ultimately, its ability to reach the intended subcutaneous tissue target [20,21,22,23]. The research is oriented towards new types of polysaccharide-based hydrogels to improve the efficiency of topical drug delivery of anti-inflammatory drugs. This category of hydrogels includes electro-conductive gels [24] and pH-sensitive gels [25]. Other branches in hydrogel research deal with implementations of preformulated drugs such as microemulsions [26], nanocomposites [27], or liposomes [28] into hydrogels structures. A lot of studies concerning IBU formulation in hydrogels investigate the properties of new types of hydrogels and compare them with commercially available products. For example, Celebi et al. [21] compared the delivery of IBU into and through the skin from novel formulations containing TEMPO-oxidized cellulose nanofibril-based (TOCN) gels to that from two conventional and commercially available products. The SC uptake and skin penetration of IBU in vitro from the novel gels and the marketed formulations were generally comparable even though the drug loading in the TOCN-based vehicles was only 20% of that in the commercial products. In vivo, the new gels appeared to enhance drug uptake into the SC following a relatively short application time, again matching the performance of the commercial formulations. The results showed that sustainable, oxidized cellulose gels may provide more efficient drug delivery into and through the skin.

Progress can also be seen in the development of new procedures for the preparation of hydrogels. As an example, Nakayama et al. [27] proposed a very simple and practical method to produce an HPMC-IBU nanocomposite gel. They mixed a methanol solution of IBU with an aqueous solution of HPMC to form a clear, uniform sol at the methanol/water proportion 7/3. When the sol was brought to dryness, nanocomposite xerogel was obtained with amorphous IBU particles of 20–50 nm dispersed in the HPMC matrix.

The modes of the in-vitro release of IBU were investigated for new types of prepared hydrogels. As an example, Montgokitikul et al. [29] prepared the pristine pectin hydrogels and conductive polymer/hydrogel blends by the solution casting using ferrous chloride and citric acid as the crosslinking agents, and IBU as the model drug and the doping agent. The in-vitro release of IBU from pectin hydrogels was found to involve 4 modes of release: Fickian diffusion; anomalous transport; case II transport; and super case II transport.

Other authors investigated new combinations of well-established gelling substances in order to improve the rheological properties and drug release of IBU. As an example, Jabeen et al. [30] synthesized and structurally characterized hydrogels composed of sodium alginate, polyethylene oxide, and acrylic acid with cyclodextrin as the hydrocolloid, prepared at different pH values, and used them for IBU delivery. The hydrogels showed significant variations in rheological properties, drug encapsulation capability, and release kinetics. The hydrogel prepared at neutral pH (pH 7) was viscoelastic, thermo-reversible, and also exhibited sol-gel transition on applying frequency and changing of the temperature. It showed the highest IBU encapsulation capacity and also optimum drug release kinetics.

In addition, the influence of IBU presence on the gelation process and the change in the composition of solution used for drug release study were investigated in the work of Weng et al. [31]. They successfully loaded a sulfonate chitosan/oxidized dextran hydrogel with different amounts of IBU. The presence of the drug accelerated the gelation process. In the static medium, the drug release rate increased with an increase in the initial drug content, and the drug release reached an equilibrium state at 24 h. Final percentages of the drug released were 93%, 84%, and 80% for the hydrogel loaded with 10%, 20%, and 30% IBU, respectively. The release in lysozyme was significantly faster than that in PBS (phosphate buffer solution) (*p* < 0.001). On day 8, the cumulative drug release percentages were about 81% and 88% for PBS and lysozyme, respectively.

Some works focused on the use of different crosslinking agents and their influences on the gel’s characteristics, toxicity, biocompatibility, and IBU release. As an example, Munster et al. [32] used solubilized dialdehyde cellulose (DAC) as an efficient crosslinking agent for poly(vinyl alcohol) (PVA) as a less toxic alternative to current synthetic crosslinking agents such as glutaraldehyde, resulting in hydrogels with comparably better characteristics. Superior mechanical properties, porosity, and surface area in comparison with analogical PVA/glutaraldehyde hydrogels were observed. Biological studies showed low toxicity and good biocompatibility of PVA/DAC hydrogels. The potential of PVA/DAC in the mesh-controlled release of biologically active compounds was investigated using IBU, rutin, and phenanthriplatin.

However, very few studies involved more than two or three types of gelling substances and compared the effect of the hydrogel type properties on drug release (i.e., comparative studies with a higher number of tested polymer hydrogels are missing). In one recent comparative study, Djekic et al. [23] formulated and evaluated a hydrogel containing IBU (5% *w*/*w*) and compared the effect of different bioadhesive polymers on their suitability for application on the skin, physical stability during the accelerated and natural aging tests, and in vitro drug release kinetics. Hydrogels were formulated with xanthan gum (XTG) 1%, NaCMC 5%, poloxamer 407 16%, and carbomer 1%. The type of the polymer significantly affected the apparent viscosity of the hydrogels and the miscibility rate with artificial sweat, their physical stability, shape, size, aggregation of the drug crystals, and degree of crystallization. The drug release in all investigated hydrogels was diffusion-controlled following the Higuchi model and sustained for 12 h, with the drug release rate and the amount of drug released depending on the polymer. The hydrogel containing 1% xanthan gum showed promising characteristics in terms of all investigated aspects.

It is well known that the gel-forming polymer concentration and structure significantly influence the flow and thixotropic parameters of the designed hydrogels as well as drug release [33,34]. Most papers dealing with the formulation of a drug into hydrogels investigated the rheological properties of different hydrogel compositions (changes in drug concentration, use of enhancers, emulsifiers, and/or other excipients). However, the influence of the own drug presence in hydrogels and the subsequent influence of rheological parameters on the drug release was evaluated only in a few articles. The rheological and texture parameters of hydrogels (e.g., Carbopol, HEC, sodium alginate, gellan gum) were influenced by the presence of the drug, their viscosity was increased [35,36]. In some cases, the drug is reported to increase the stiffness of the polymer network. The rheological parameters seem to be more precise and sensitive to some changes in the mechanical properties of the gels [36]. However, the data are scarce and a complex view on this topic is currently missing.

The above-mentioned studies highlight the importance of polysaccharide-based hydrogels in the topical administration of NSAIDs such as IBU. For proper use of individual hydrogels or their mutual combinations in a new dosage form development, a comprehensive comparative study providing an exact view on differences in their rheological, texture, and drug release properties is a prerequisite. To our best knowledge, however, an extensive comparative study of IBU in vitro diffusion through a semi-permeable membrane from a variety of different polysaccharide hydrogels and evaluation of their properties is missing. Therefore, our work aimed to solve this issue via investigation of large groups of hydrogels based on 11 different natural and semi-synthetic polysaccharide polymers, namely methylcellulose (MC), hydroxypropylmethylcellulose (HPMC), hydroxyethylcellulose (HEC), carboxymethylcellulose (CMC), sodium salt of carboxymethylcellulose (NaCMC), tragacanth (TRG), carrageenan (CRG), chitosan derived from crab (CSc), chitosan derived from shrimps (CSs), high molecular weight chitosan (CS HMW), and xanthan gum (XTG). Rheological and texture properties along with the in vitro diffusion profiles of model drug IBU were evaluated and compared for each type and several concentrations of the hydrogel. Drug release kinetics was evaluated for five kinetic models, i.e., zero-order, first-order, Higuchi, Korsmeyer–Peppas, and Hixon–Cromwell. To demonstrate the possibility for a deliberate modification of hydrogel properties, three hydrogels based on the combinations of two different polysaccharides were developed and compared for differences in their IBU diffusion profiles. Considering drug release data corrected for differing viscosities, the effect of different chemical structures of particular hydrogels was investigated. From the practical point of view, the hydrogel appropriate for the dermal application (i.e., providing favorable rheological and texture properties along with the highest drug release) was selected from a studied series of polysaccharide-based hydrogels.

## 2. Results and Discussion

Hydrogels were prepared from 11 different polysaccharide gelling substances (MC, HPMC, HEC, CMC, NaCMC, TRG, CRG, XTG, and three CS analogs including CSc, CSs, and CS HMW) differing by their polarities and charges to study and compare the influence of these parameters on their gel properties and IBU diffusion profiles. From this point of view, the hydrogels were divided into three groups, namely (i) neutral including MC, HPMC, HEC, (ii) anionic including CMC, NaCMC, TRG, CRG, XTG, and (iii) cationic including CSc, CSs, and CS HMW; for basic structural information of the used polymers, see Table A1 in Appendix A.

The hydrogels were prepared from each polymer at 4 individually chosen concentrations to obtain 4 preparations with different viscosities—from low viscosity to high viscosity, together 44 studied hydrogels. In the first step, rheological measurements were carried out for all prepared hydrogels to assess their rheological behavior and viscosity. In the second step, the texture properties of the hydrogels selected in the first step were tested. In the third step, in vitro diffusion of IBU from the hydrogels (selected in steps 1 and 2) through a semi-permeable membrane was evaluated. In the fourth step, drug release from hydrogels composed of two different polymers was tested and compared with a corresponding single polymer-hydrogel.

The value of this study lies in the fact that a relatively large group of the polysaccharide hydrogels differing in their physical-chemical properties was taken into the consideration for the measurements in (i) one laboratory, on (ii) one set of equipment, and with (iii) the same chemical substances. In this way, highly reliable experimental data (rheological, texture, kinetic, etc.) could be obtained and relevant conclusions could be drawn. Indeed, a lot of work is reported in the literature on polysaccharides for developing hydrogels. According to the best of our knowledge, however, such comprehensive evaluation and comparison within a large group of the polysaccharide hydrogels demonstrated on the IBU release model have not been carried out so far.

### 2.1. Rheological Parameters

**Figure 1:**
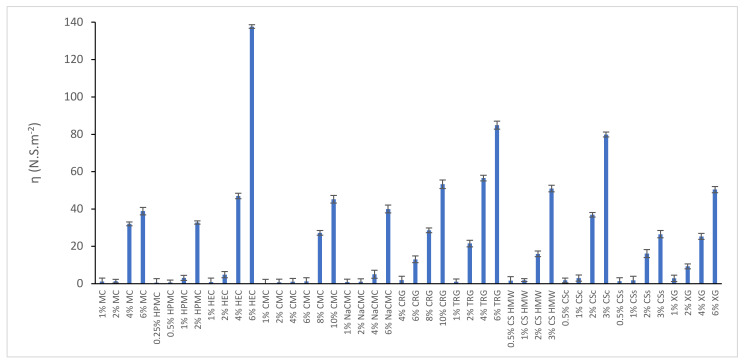
Structural viscosity of all prepared polysaccharide-based hydrogels at a shear rate of 6.45 s^−1^.

**Figure A1:**
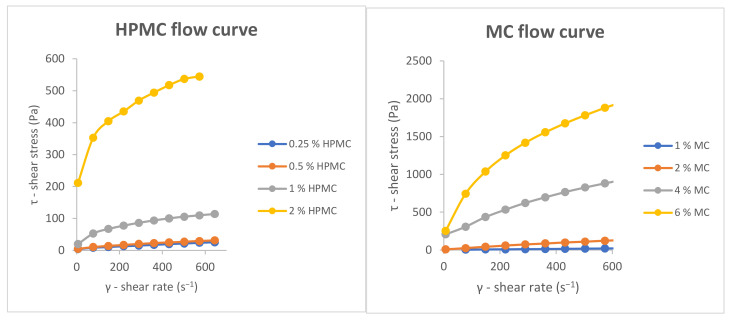

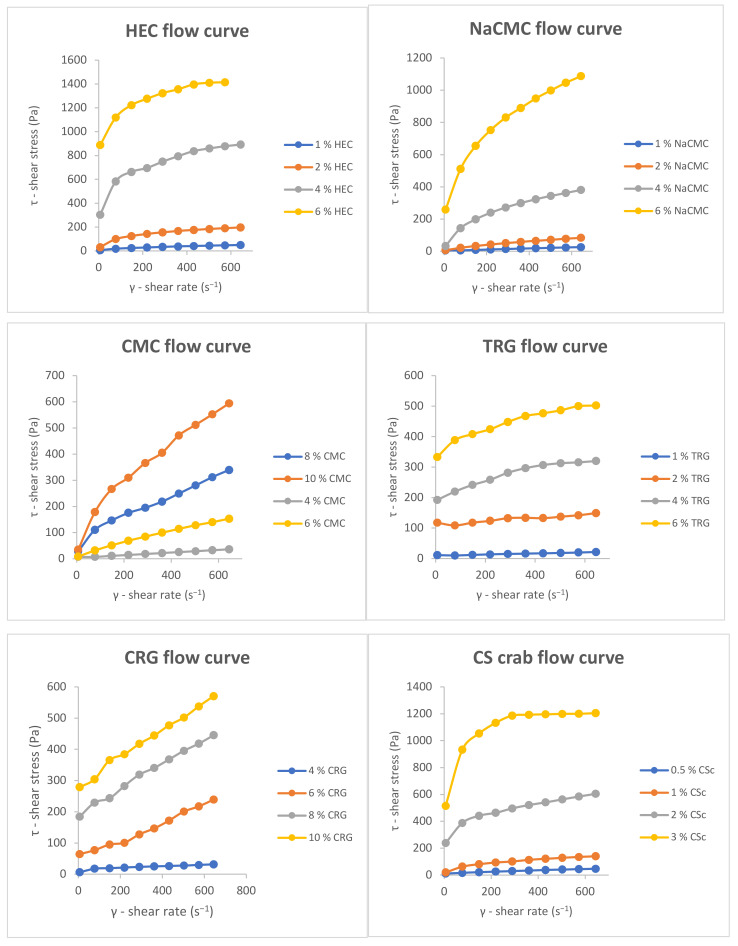

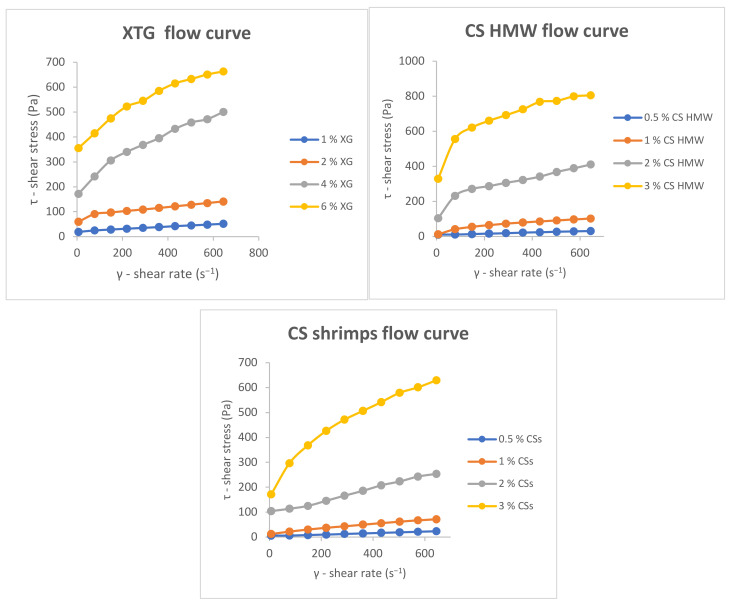
Flow curves of polysaccharide-based hydrogels of different concentrations at increasing shear rate.

Rheological curves for all prepared polysaccharide-based hydrogels were evaluated and the structural viscosity at the minimum shear rate (6.45 s^−1^) was compared as is illustrated in Figure 1. At this shear rate, 6% HEC hydrogel has the highest structural viscosity. Flow curves of all hydrogels at increasing shear rates are illustrated in Figure A1 in Appendix A. As was expected, the flow curves changed from nearly Newtonian flow (in low concentrated hydrogels) to pseudo-plastic or plastic flow with increasing content of gelling substance. This knowledge of rheological properties helped to choose suitable hydrogels for topical (dermal) application. Based on values of structural viscosity, hydrogels were divided in three categories, i.e., low-viscous (<10.0 N.S m^−2^), medium-viscous (10.0–50.0 N.S m^−2^), and high-viscous (>50.0 N.S m^−2^). Due to their favorable properties for topical applications, two later categories (including 6% MC, 2% HPMC, 4% and 6% HEC, 4% and 6% NaCMC, 8% and 10% CMC, 2%, 4% and 6% TRG, and 8% and 10% CRG, 4% and 6% TXG, 2% and 3% CSc, 2% and 3% CSs, 2% and 3% CS HMW) were further studied for texture properties and IBU release.

**Figure 2:**
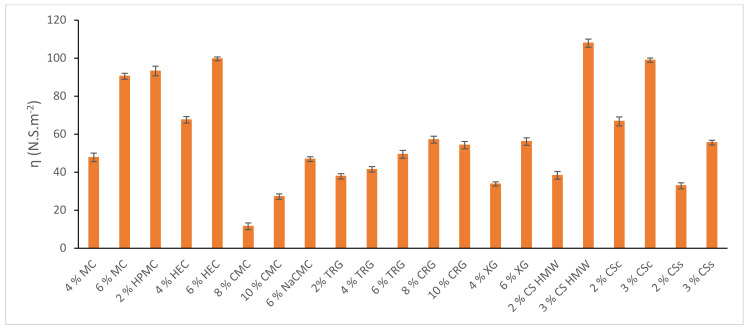
Structural viscosity of chosen polysaccharide-based hydrogels containing IBU at a shear rate of 6.45 s^−1^.

**Figure A2:**
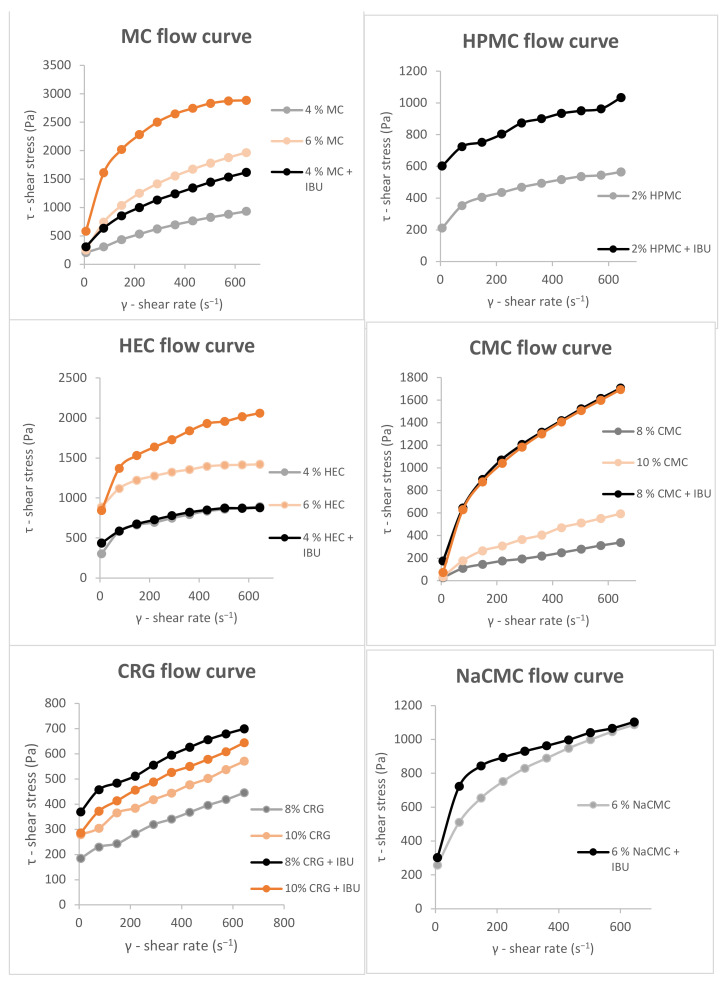

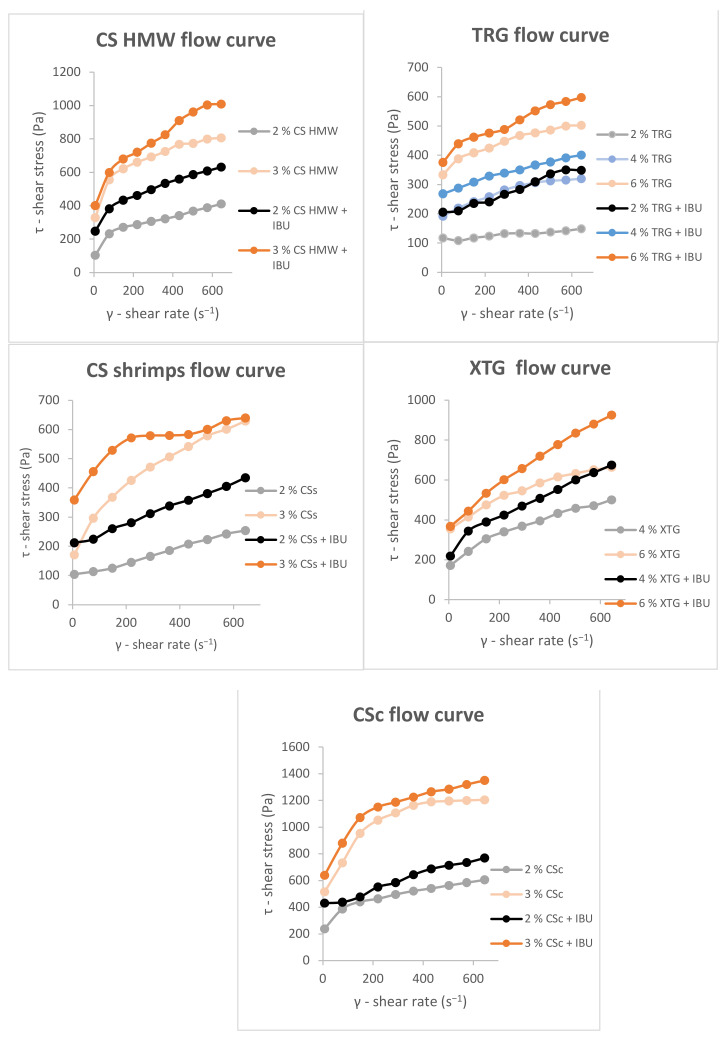
Flow curves of polysaccharide-based hydrogels of chosen concentrations without IBU and containing IBU at increasing shear rate.

Rheological curves were evaluated and compared within two series of hydrogels, one containing IBU and another one without IBU. Results are illustrated in Figure A2 in Appendix A. In all hydrogels, the presence of IBU led to an increase in shear stress (Pa). The structural viscosity of IBU-containing hydrogels at the minimum shear rate (6.45 s^−1^) was illustrated in Figure 2. The structural viscosity ranged in the interval of 11.49–107.91 N.S m^−2^, therefore all hydrogels belong to the medium- and high-viscous hydrogels. All tested IBU-containing hydrogels are principally suitable for topical application.

### 2.2. Texture Parameters

**Figure 3:**
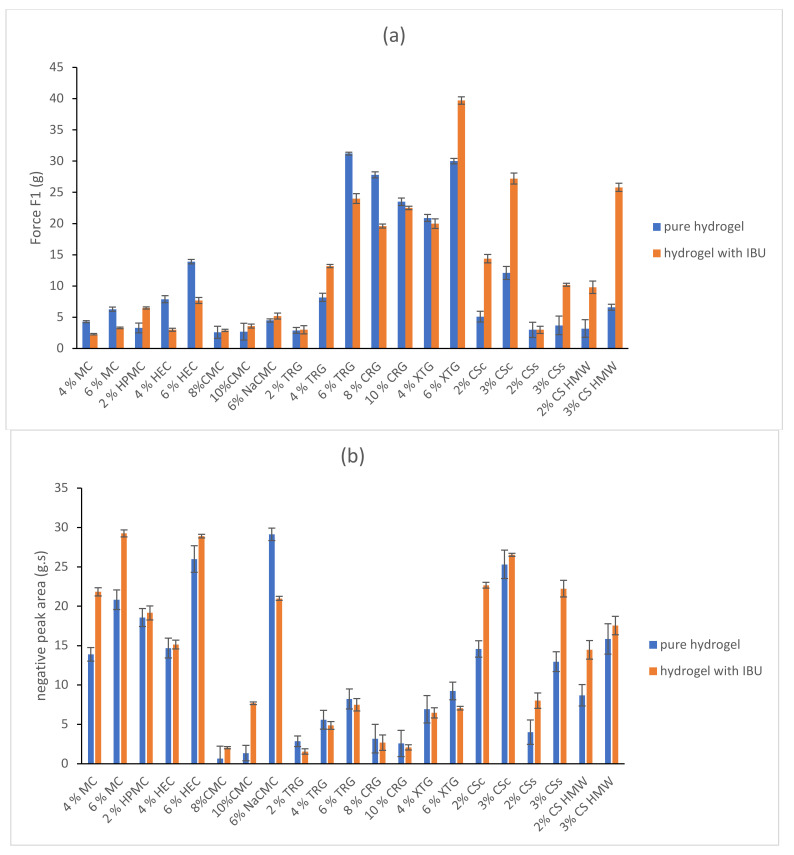

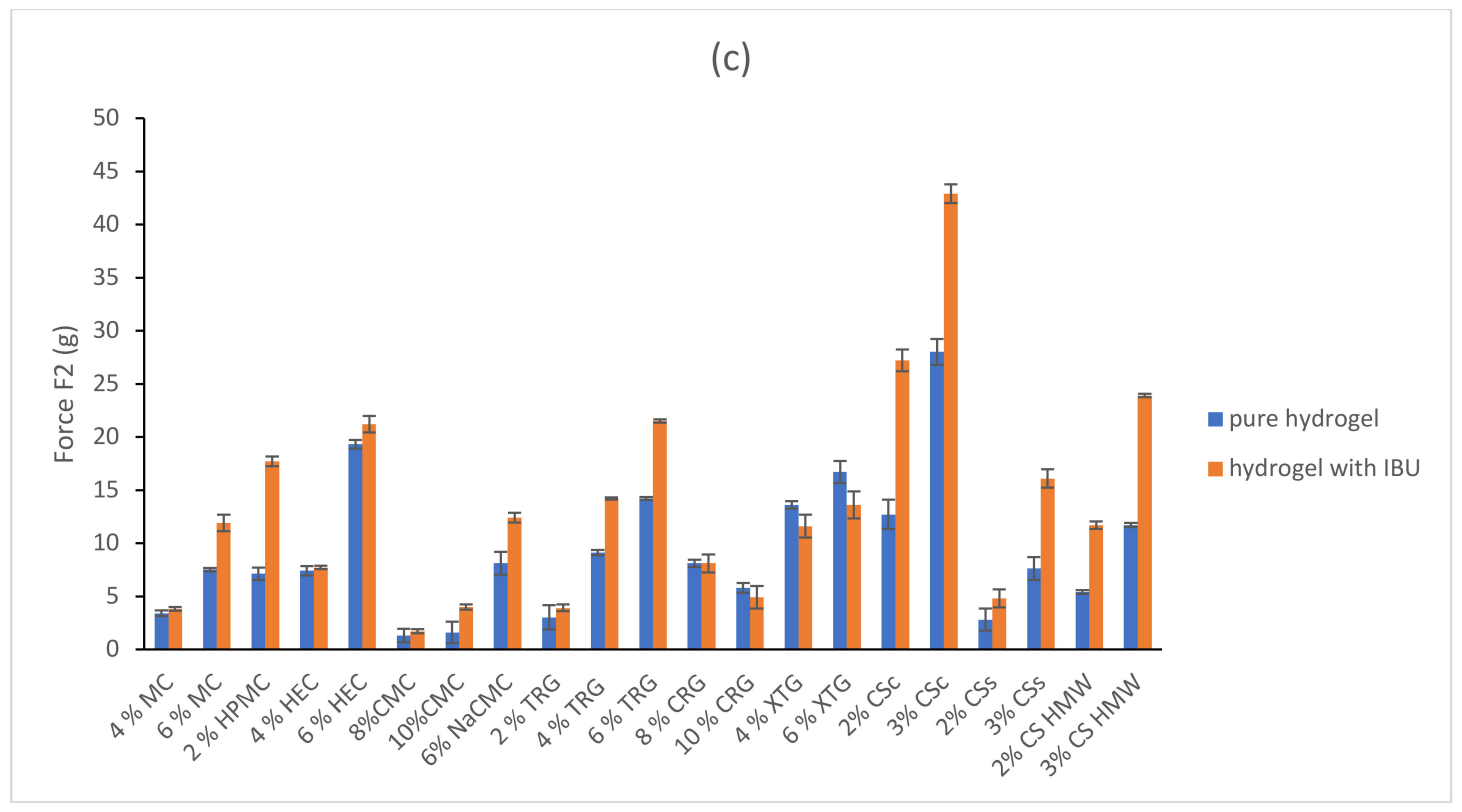
Comparison of hardness (**a**), adhesiveness (**b**), and minimal retracting force (**c**) of chosen hydrogels without IBU and containing IBU.

The texture profile analysis was used to investigate the effect of the concentration of the gelling substance on the mechanical properties of the resulting hydrogel. Hardness (maximal compressing force), adhesiveness, and minimal retracting force were evaluated by the texture profile analysis of the hydrogels selected in the first step (after rheological testing). The results are shown in Figure 3.

The hardness (Figure 3a) ranged in the interval of 2.6 g (8% CMC)–31.2 g (6% TRG) and increased with increasing concentration. Both XTG hydrogels (4% and 6%) showed a relatively high hardness (20.9 and 30.0 g, respectively). In comparison, the hardness of CS hydrogels was considerably lower. When comparing the hardness of three types of CS hydrogels, CSc showed the highest values (12.1 and 5.1 g) for both studied concentrations (3 and 2%, respectively). Generally, the hardness of CS gel was increasing in order CSs, CS HMW, and CSc.

The adhesiveness (Figure 3b) ranged in the interval of 0.65 (8% CMC)–29.1 (6% NaCMC) g.s. Good adhesiveness was observed in 6% HEC (26.0), 3% CSc (25.3), and 6% MC hydrogel (20.8) g.s. In all cases except for CRG, the adhesiveness increased with the increasing concentration of the gelling substance.

The minimal retracting force (Figure 3c) ranged in the interval of 1.3 (8% CMC)–28.0 (3% CSc) g. The highest value of minimal retracting force was generally observed in CSc hydrogels (12.7 and 28.0 g), XTG gels (13.6 and 16.7 g), and HEC hydrogels (7.4 and 19.3 g).

A topical preparation should be viscous enough to be taken out of the container and have semi-solid character, easily spreadable on the skin, and possess suitable adhesiveness to stay in contact with the skin. When considering the hardness and adhesiveness to be suitable for topical administration, the most promising tested hydrogels were 6% MC, 4% and 6% HEC, 4% and 6% TRG, 4% and 6% XTG, 2% and 3% CSc and 3% CS HMW with hardness > 5 g, adhesiveness > 5 g.s. and minimal retracting force > 5 g.

**Figure 4:**
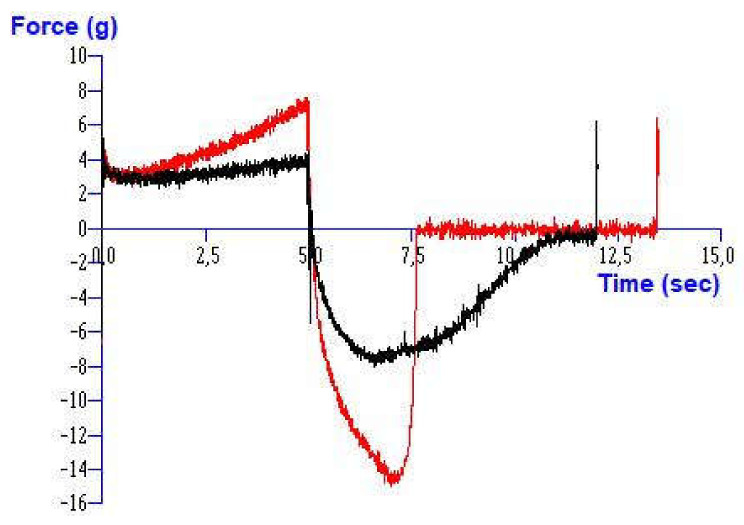
Texture curve (dependence of compressing force on time) of pure 6% hydrogel of NaCMC (black curve) and 6% NaCMC containing 5% of ibuprofen (red curve).

To evaluate the influence of IBU presence in the hydrogels on their texture properties, all the selected hydrogels were formulated with a 5% concentration of IBU. An illustrative example of texture curve (dependence of compressing force on time) of the blank 6% NaCMC hydrogel and 6% NaCMC containing 5% of IBU is illustrated in Figure 4. A significant influence of IBU content on the hardness of hydrogel (increase in maximum compressing force) and the minimal retracting force (increase) was observed. Moreover, the shape of the negative peak and area under the curve (AUC) was changed (decreased) as a result too.

A 5% IBU content in the polysaccharide-based hydrogels changed their texture properties differently as is illustrated in Figure 3. In the majority of hydrogels, the IBU formulation led to an increase of hardness (2% HPMC, 8% and 10% CMC, 6% NaCMC, 2%, 4% and 6% TRG, 4% and 6% XTG, 2% and 3% CSc, 2% and 3% CSs, and 2% and 3% CS HMW), but in some minor cases, the hardness was decreased (4% and 6% MC, 4% and 6% HEC, 8% and 10% CRG). The adhesiveness was increased with the presence of IBU in the hydrogels composed of MC, HEC, CMC, XTG, and all types of CS. On the other hand, the adhesiveness of HPMC, NaCMC, TRG, CRG, and XTG hydrogels was decreased when containing IBU. The minimal retracting force in the majority of the hydrogels increased when implementing IBU. The CRG and XTG hydrogels were found to be the exceptions where the presence of IBU caused a decrease in minimal retracting force. In the tested IBU-containing hydrogels, the most promising for dermal application (with chosen parameters: hardness > 5 g, adhesiveness > 5 g.s. and minimal retracting force > 5 g) were 2% HPMC, 6% HEC, 6% NaCMC, 6% TRG, 4% and 6% XTG, 2% and 3% CSc, 3% CSs, and 2% and 3% CS HMW.

Indeed, according to the best of our knowledge, there are no published articles dealing with the influence of IBU content on either the rheological or texture properties of studied polysaccharide hydrogels. Nonetheless, similarly to our comparative study (i.e., hydrogel properties with or without IBU), there have been published several works dealing with some other similar (hydrophobic with a carboxylic group) anti-inflammatory drugs such as ketoprofen or indomethacin. The content of IBU increased the shear stress of hydrogels [34,37]. All our hydrogels were physically crosslinked. Physically crosslinked hydrogels are fragile with low mechanical integrity and high degradation rate [35]. In agreement with our observation with IBU, the authors reported in their works that the content of ketoprofen or indomethacin increased the shear stress of various hydrogels (Carbopol, HEC, sodium alginate, gellan gum). Analogically, the drug mesalazine showed the ability to increase the stiffness of the polymer network (acyl gellan gum) [36], as it was observed with IBU and the studied polysaccharide gels in our work.

From our chosen hydrogels, the best composition for topical applications, compromising within the parameters of viscosity, rheological curves, hardness, adhesiveness, and minimal retracting force, were 6% TRG, 6% XTG, 2% and 3% CSc, and 3% CS HMW.

### 2.3. Drug Release Behavior in Hydrogels Composed of a Single Polymer

**Figure 5:**
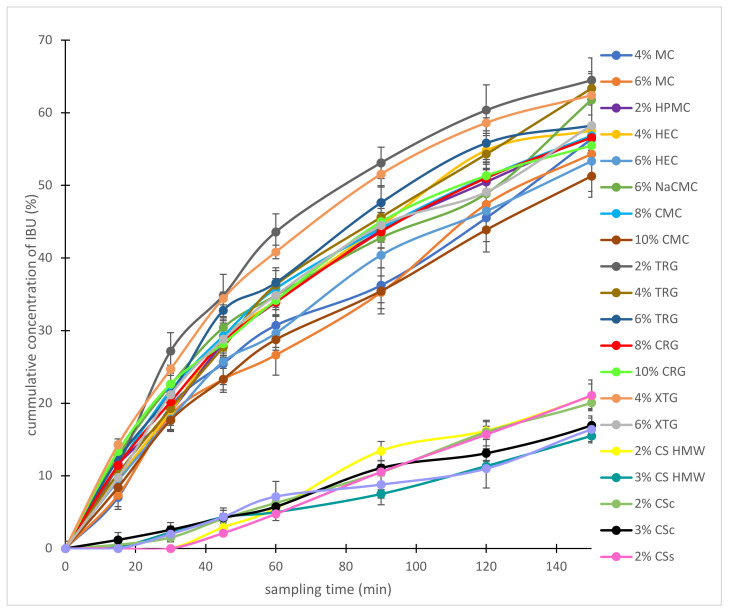
The cumulative amount of ibuprofen released from chosen hydrogels with time.

**Figure A3:**
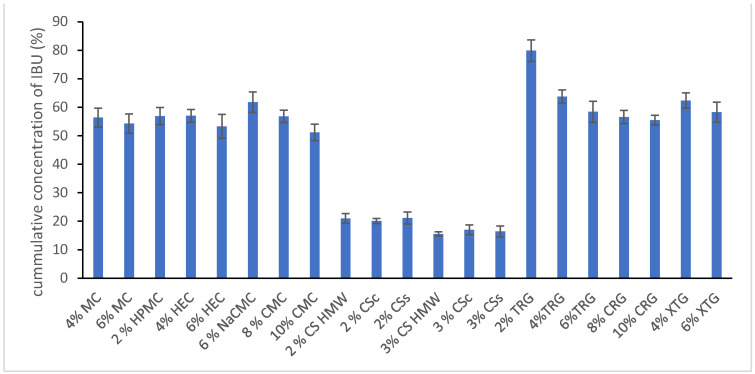
Comparison of the cumulative amount of ibuprofen (%) released from chosen hydrogels after 150 min.

**Figure A4:**
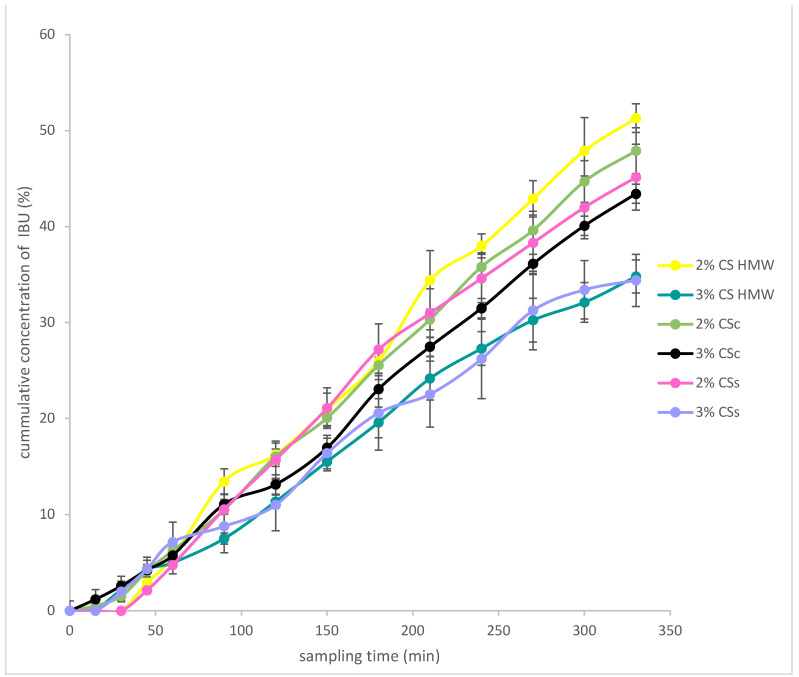
The cumulative amount of ibuprofen released from chosen chitosan hydrogels with time.

**Figure A5:**
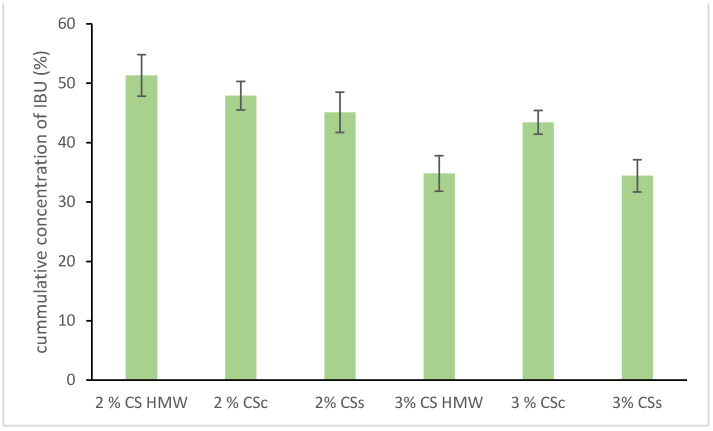
Comparison of the cumulative amount of ibuprofen (%) released from CS hydrogels after 330 min.

Drug release by in vitro diffusion of IBU from chosen hydrogels through semi-permeable membrane was evaluated using Franz cells and the resulting diffusion dependences are illustrated in Figure 5. The amount of released IBU after 150 min was compared for individual hydrogels and the results are shown in Figure A3 in Appendix A. The amount of released IBU increased in order 3% CS HMW (15.5%), 3% CSs (16.9%), 3% CSc (17.0%), 2% CS HMW (20.9%), 2% CSc (20.1%), 2% CSs (21.0%), 10% CMC (51.2%), 4% MC (52.5%), 6% HEC (53.3%), 6% MC (54.3%), 10% CRG (55.5%), 8% CRG (56.6%), 8% CMC (56.8%), 2% HPMC (56.9%), 4% HEC (57.0%), 6% TRG (58.2%), 6% XTG (58.3%), 6% NaCMC (61.8%), 4% XTG (62.4%), 4% TRG (63.4), and 2% TRG (64.5%). The diffusion from CS hydrogels was much slower than from other polysaccharide-based hydrogels. Therefore, the release of IBU was carried away for another 180 min. The results are illustrated in Figure A4 in Appendix A. The amount of released IBU after 330 min was compared for individual CS hydrogels and the data are graphically depicted in Figure A5 in Appendix A. The amount of released IBU increased in order was 3% CSs (34.4%), 3% CS HMW (34.8%), 3% CSc (43.4%), 2% CSs (45.1%), and 2% CSc (47.9%). 2% CS HMW showed the fastest release in 330 min (51.3%).

**Figure 6:**
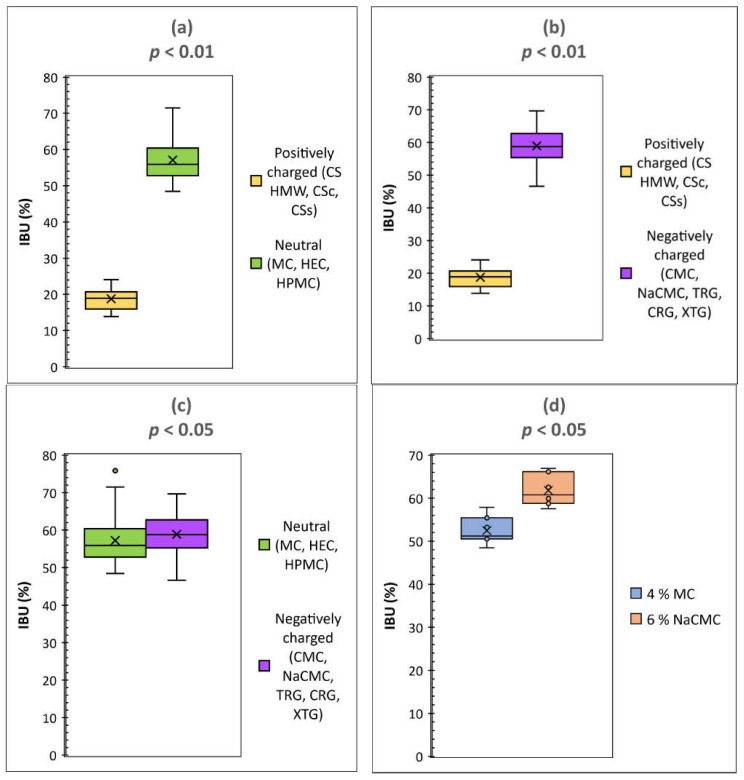
The statistical evaluation (Students’s *t*-test) of IBU release for particular groups of the hydrogels, characteristic by their charge. Comparison of (**a**) cationic hydrogels with neutral ones, *p* = 0.000, (**b**) cationic hydrogels with anionic ones, *p* = 0.000, (**c**) neutral hydrogels with anionic ones, *p* = 0.044, (**d**) neutral hydrogel (4% MC) with anionic one (6% NaCMC), having similar viscosities of 32.05 and 40.04 N.S m^−2^ at shear rate 6.45 s^−1^, respectively, *p* = 0.012.

As expected, the release of IBU was faster from low concentrated hydrogels due to their lower viscosity when comparing the hydrogels based on the same polysaccharide differing in concentrations. Similar results showed, e.g., cross-linked CS hydrogels containing diclofenac according to Iglesias et al. [25]. The above-mentioned data also clearly indicated more or less significant differences between the hydrogels with different polysaccharide structures. The statistical evaluation (Student’s *t*-test) of IBU release for particular groups of the hydrogels, charactered by their charge, is shown in Figure 6. Significant differences (*p* < 0.01) in IBU diffusion were statistically confirmed between the groups of the hydrogels composed of the positively charged polysaccharides on one side and neutral (Figure 6a) or negatively charged ones (Figure 6b) on the other side. This behavior can be logically explained via stabilization of CS-IBU associates by attractive Coulomb interactions, and, thus, a slower release of IBU from the CS hydrogels. Similar to our interpretation of the effect of ionic interactions between CS and IBU, Quandil et al. [38] found that complexation of IBU with CS involves ionic interaction between the ammonium group of CS and the carboxylate anion of IBU. Puttipipatkhachorn et al. [39] reported the drug-polymer interaction affected the release of salicylic acid from the CS films and resulting in sustained release action. They summarized that CS could interact via its amino group with negatively charged (acidic) drugs when incorporated into films and this might affect the drug release characteristics. Bernkop-Schnürch and Dünnhaupt [40] demonstrated that using polyanionic drugs, the interactions between CS and the therapeutic agent are more pronounced, and based on an ionic cross-linking in addition, even stable complexes are formed from which the drug can be released even over a more prolonged time. Tajmir-Riahi et al. [41] reported that drug–CS complexation occurred via hydrophobic and hydrophilic contacts as well as H–bonding network. Su et al. [42] reported that Coulombic interactions, van der Waals force, H–bonding, etc. could be present when interacting CS with cefazolin sodium. When comparing the groups of hydrogels composed of negatively charged and neutral polysaccharides, differences in IBU release seemed to be considerably lower (*p* < 0.05) but they are still statistically significant (Figure 6c). In order to highlight the importance of different physical-chemical interactions (i.e., ionic vs. nonionic) of the compared neutral and ionic hydrogels on IBU release velocity, the comparison was carried out at their similar viscosities (to minimize the effect of viscosity on the release velocity). The statistical significance of the release data of two hydrogels possessing similar viscosities, the neutral and negatively charged one, is illustrated in Figure 6d. In this way, it was possible to assess the effect of different chemical structures/interactions in the compared hydrogels on IBU release properly, showing, in general, a higher IBU diffusion from anionic hydrogels in comparison with the neutral ones (probably due to the repulsive Coulomb forces between negatively charged groups in the ionic polymers and negatively charged (carboxylate) group of IBU). Similar to our interpretation of the effect of repulsive ionic interactions between the tested anionic hydrogels (NaCMC, CMC, TRG, CRG, XTG) and IBU on the drug diffusion, Veeramachineni et al. [43] reported the effect of ionic repulsion between the anionic carboxymethyl TRG and anionic diclofenac sodium on drug entrapment efficiency.

Drug release kinetics were compared through the coefficient of determination (R^2^) expressed for the regression line of five kinetic models, and flux (Jss) and permeation coefficient (K_p_) were calculated, see data in Table 1. The values indicate that the drug release of polysaccharide-based hydrogels during in vitro diffusion studies follows mostly the Higuchi model (in the cases of 6% MC and 2% HPMC first-order kinetic), whereas drug release from CS hydrogels follows zero-order kinetic. These results were in agreement with Dejkic et al. [23]. They investigated IBU release from hydrogels from XTG and NaCMC, although in different concentrations and using isopropyl alcohol as a solvent for IBU. Generally, the drug release rate, which follows zero-order kinetics, is independent of the drug concentration, i.e., it is not influenced by increasing or decreasing drug concentration. Conversely, when the drug is released by first-order kinetics, the drug permeation rate is greatly affected by the drug concentration. In contrast, the Higuchi model characterizes the kinetics of drug release from sustained release formulations or transdermal systems [33]. Therefore, it may be concluded that the IBU release from CS hydrogels is sustained without initial burst release as suggested by other works [44]. The permeation coefficient ranges from 0.721 to 2.420 cm.h^−1^, the highest in 4% and 2% TRG hydrogels 2.420 and 2.416 cm.h^−1^, respectively. CS hydrogels showed minimal permeation coefficient ranging from 0.721 cm.h^−1^ (3% CSs) to 1.230 cm.h^−1^ (2% CS HMW).

Summarizing, the effect of ion–ion interactions between the drug and hydrogel (selected from one of three subgroups of the studied polymers differing in their charges) on the drug release profile was clearly demonstrated and supported by statistical analysis of data. The knowledge on the relationship of such effect to the given drug–polymer system, as demonstrated in our work, has a practical value in the creation of novel drug delivery systems as for topical so as for other kinds of applications. Thus, information from this study will be essential, e.g., in the development of combined hydrogel systems with modified rheological and texture properties and drug release profiles as well as formulations of other structurally related drugs.

When considering potential topical applicability of the single polymer-based hydrogels, criteria for the choice of proper hydrogel include fast drug release along with appropriate rheological and texture properties of the hydrogel dosage form. Although 2% hydrogel of TRG showed the fastest release of IBU, 4% hydrogel of XTG was chosen as optimal for topical dermal application because of its better adhesiveness, hardness, and viscosity, along with a still relatively fast release of IBU.

### 2.4. Drug Release Behavior in Hydrogels Composed of Combined Polymers

From the practical point of view, individual CS hydrogels are less suitable for topical applications of IBU because of their slow release (only 21% of IBU was released as a maximum in 150 min). On the other hand, the prolonged release of IBU can be utilized advantageously for other than topical applications (e.g., peroral) demanding a lower dose to be released per time unit. Hydrogels providing a slow release also can serve as a favorable base for the creation of hydrogel preparations offering specific release profiles, e.g., due to a combination of different gelling agents (polymers). To select a proper combination for a given purpose, the knowledge of properties and behavior of individual hydrogels as well as drug release kinetics, as described above in Section 2.1, Section 2.2 and Section 2.3, is a prerequisite.

**Figure 7:**
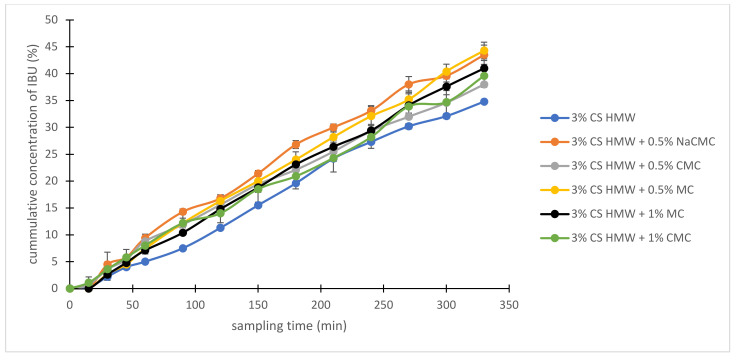
The cumulative amount of IBU released from chosen combinations of hydrogels with time.

**Figure 8:**
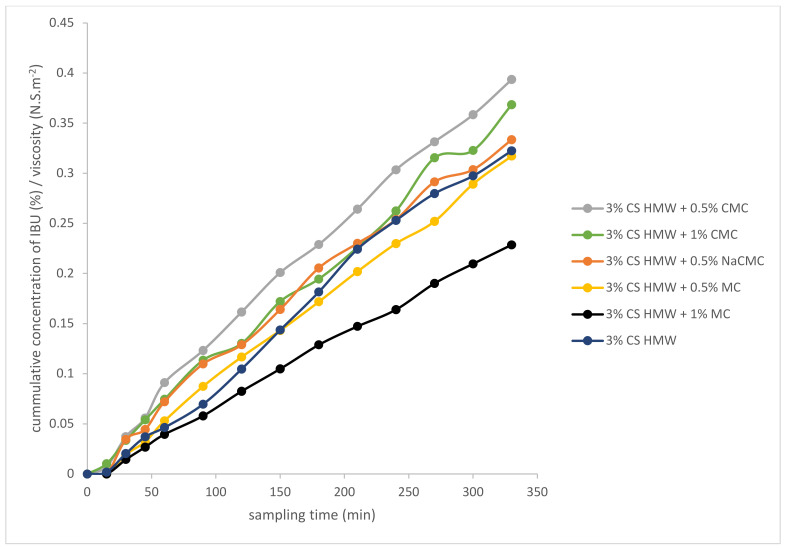
The cumulative amount of IBU released from chosen combinations of hydrogels in time, related to viscosity.

As illustrative examples, we studied three combinations of polysaccharide hydrogels for IBU diffusion: (i) cationic hydrogel with ionic (anionic) one, (ii) cationic hydrogel with ionizable (anionic) one, and (iii) cationic hydrogel with a neutral one. For this, 3% CS HMW was combined with 0.5% and 1% NaCMC (i), CMC (ii), and MC (iii). This study was designed to evaluate the role of the ionic and non-ionic interactions between combined polymers and anionic drugs (represented by IBU) on its diffusion profiles. The hydrogel containing 1% NaCMC showed very high viscosity (150.3 N.S m^−2^) and was not suitable for dermal application. The IBU diffusion from the other combined hydrogels is illustrated in Figure 7. To eliminate the influence of hydrogel viscosity and see a net effect of different structures/interactions of the tested combined hydrogels on the drug diffusion, the drug release data were related to (corrected on) the viscosity of the prepared gels, and the resulting (corrected) curves are shown in Figure 8. A combination of cationic hydrogel (CS) with the neutral one (MC) showed the slowest IBU release, it was even slower than in the case of CS alone. It can be explained simply by an increased number of free functional groups of the polymers offering additional interactions/binding (mainly H–bonds) with IBU. On the other hand, a combination of cationic hydrogel (CS) with anionic ones (NaCMC and CMC) speeds up the IBU release. In this case, however, the situation can be more complicated. The IBU diffusion can be influenced by several mechanisms including ionic crosslink of the polymers via their oppositely charged groups, attraction of IBU via free (non-crosslinked) amino groups of CS, repulsion of IBU via free (non-crosslinked) carboxylate groups of NaCMC or CMC, and, obviously, binding via several electroneutral functional groups of NaCMC or CMC (mainly through H–bonds).

The CS hydrogel, although not suitable for a topical application of IBU due to very slow drug release, was presented as a highly effective tool for modifying fast drug release profiles of neutral or oppositely charged polysaccharide hydrogels. This knowledge can be advantageously utilized in developing hydrogel systems aimed at other than topical applications. It can be generalized that a combination of CS with neutral or oppositely charged polymers can be used for effective control of drug release towards higher or lower released amounts per time unit.

## 3. Concluding Remarks

In this comparative study, rheological and texture properties of 11 polysaccharide-based hydrogels were comprehensively investigated along with the in vitro release of model drug ibuprofen (IBU). At first, the rheological properties of the hydrogels as dependencies of shear stress on the shear rate were evaluated to recognize the type of flow. Based on viscosity values, three categories of hydrogels were selected, i.e., low-, medium- and high-viscous and two later categories (including 4% and 6% HEC, 4% and 6% MC, 2% HPMC, 6%Na CMC, 8% and 10% CMC, 2%, 4% and 6% TRG, 8% and 10% CRG, 2% and 3% CSc, CSs and CS HMW, and 4% and 6% XTG) were further studied for texture properties. In the second step, hardness, adhesiveness, and minimal retracting force, were evaluated by texture profile analysis. The adhesiveness ranged in the interval of 0.65–29.1 g.s. In the third step, the release of IBU from these individual hydrogels was evaluated. Drug release kinetics were compared through the coefficient of determination (R^2^) expressed for the regression line of five kinetic models. The values indicated that the drug release of polysaccharide-based hydrogels during in vitro diffusion studies follows mostly the Higuchi model (in the cases of 6% MC and 2% HPMC first-order kinetic), whereas drug release from CS hydrogels follows zero-order kinetic. The amount of released IBU in 150 min increased in order 3% CS HMW (15.5%), 3% CSs (16.9%), 3% CSc (17.0%), 2% CS HMW (20.9%), 2% CSc (20.1%), 2% CSs (21.0%), 10% CMC (51.2%), 4% MC (52.5%), 6% HEC (53.3%), 6% MC (54.3%), 10% CRG (55.5%), 8% CRG (56.6%), 8% CMC (56.8%), 2% HPMC (56.9%), 4% HEC (57.0%), 6% TRG (58.2%), 6% XTG (58.3%), 6% NaCMC (61.8%), 4% XTG (62.4%), 4% TRG (63.4), and 2% TRG (64.5%).

Significant differences in IBU diffusion were found and statistically confirmed among the groups of the hydrogels composed of positively charged, neutral, or negatively charged polysaccharides. Here, differences in provided interactions (mainly attractive/repulsive Coulomb forces) of the ionic polymers with the formulated anionic drug played a key role in different IBU release profiles, as supported by statistical analysis (*p* < 0.01 or *p* < 0.05 in Student’s *t*-test). The knowledge on provided interactions can be utilized not only when formulating one drug, but also when simultaneously formulating several drugs with different character demanding their different release kinetics.

From the practical point of view, 4% hydrogel of anionic XTG was chosen as optimal for its dermal application. It is a compromise between the highest releases of IBU and the best adhesiveness, hardness, and viscosity when considering a studied series of polysaccharide-based hydrogels.

The knowledge on rheological and texture properties as well as drug release kinetic profiles of individual polysaccharide hydrogels, as comprehensively presented in this work, can also be advantageously utilized in the creation of various hydrogel preparations with modified drug release profiles suitable for particular drug delivery purposes. As model examples, the hydrogels providing prolonged and fast release of IBU (differing in their charges) were mutually combined and the diffusion profiles of IBU were demonstrated with respect to the contribution of viscosity and interactions on their differences. The CS hydrogel, although not suitable for a topical application of IBU due to very slow drug release, was presented as a highly effective tool for modifying fast drug release profiles of neutral or oppositely charged polysaccharide hydrogels. Hence, for future studies, when considering the formulation of a group of hydrophobic drugs possessing a negatively charged functional group (as demonstrated via IBU herein), CS can be advantageously utilized as a basis for designing new (e.g., combined polymer) systems for controlled drug release mainly prolonged or sustained.

## 4. Materials and Methods

Chemicals were purchased: MC—Ph. Eur. 9.0 (Dr. Molar Chemicals KFT, Halásztelek, Hungary), HPMC (Dr. Kulich Pharma, s.r.o., Hradec Králové, Czech Republic), HEC—Natrosol 250 (Hercules Aqualon, Wilmington, DE, USA), CMC (Dr. Kulich Pharma, s.r.o., Hradec Králové, Czech Republic), NaCMC (Dr. Kulich Pharma, s.r.o., Hradec Králové, Czech Republic), TRG (Alfa Aesar, Heysham, UK), CRG (Sigma Aldrich, St. Louis, MO, USA), XTG (Sigma Aldrich, St. Louis, MO, USA), CS HMW (Sigma Aldrich, St. Louis, MO, USA), CSc (Sigma Aldrich, St. Louis, MO, USA), CSs (Sigma Aldrich, St. Louis, MO, USA), deionized water (Biosan, Vrhnika, Slovenia), and ibuprofen (Fagron a.s., Olomouc, Czech Republic).

### 4.1. Preparation of Hydrogels

A measure of 10.0 g of hydrogels were prepared by dispergation in deionized water with 4 mL of 10% solution of NaHCO_3_: MC (1, 2, 4 and 6% *w*/*w*), HPMC (0.25, 0.5, 1 and 2% *w*/*w*), HEC (1, 2, 4 and 6% *w*/*w*), CMC (4, 6, 8 and 10% *w*/*w*), NaCMC (1, 2, 4 and 6% *w*/*w*), TRG (1, 2, 4 and 6% *w*/*w*), CRG (4, 6, 8 and 10% *w*/*w*), and XTG (1, 2, 4 and 6% *w*/*w*), at proper temperature. Formed hydrogels stayed in the refrigerator for at least 24 h to swell and become clear.

### 4.2. Preparation of Hydrogels Containing Ibuprofen

Hydrogels (10.0 g) of chosen concentrations containing 5% ibuprofen and 4 mL of 10% NaHCO_3_ (for IBU solubilization) were prepared.

Combined hydrogels were prepared in the following way: 5% IBU dissolved in concentrated ethanol was mixed with 3% CS HMW and 0.5% or 1% MC, CMC, or NaCMC in 2% acetic acid to form a hydrogel.

### 4.3. Rheological Measurements

Rheological experiments were performed to examine the viscous and elastic properties of prepared hydrogels. Samples were analyzed using a controlled rate rotational rotation rheometer (rheometer/rotation viscosimeter Rheolab QC ANTON PAAR^®^, Graz, Austria) at 25 °C. During increasing shear rate and successive decreasing shear rate, parameters such as viscosity and shear stress were noted.

### 4.4. Texture Analysis

Samples of hydrogels (tempered to 25 °C) were studied by a texture analyzer (TA.XT.Plus, Stable Micro Systems, Godalming, UK) with compression. The hydrogel’s parameters such as hardness, cohesiveness, and adhesiveness were determined from the resultant force-time plot. Cohesiveness is defined as the work required to deform the hydrogel in the downward movement of the probe, adhesiveness is the work required to withdraw the probe from the hydrogel. Hardness is the maximum compressing force.

### 4.5. In Vitro Drug Release

The release of IBU from chosen polysaccharide-based hydrogels was determined by using Franz diffusion cells (laboratory-made in Dpt. Of Galenic Pharmacy, Faculty of Pharmacy Comenius University Bratislava, Slovak Republic), (7 parallel measurements) with dialysis cellulose membrane. The artificial membrane Nephrophan (VEB Filmfabrik, Wolfen, Germany) is a selective cellulose acetate membrane, microporous, and highly hydrophilic polymeric filter with a pore diameter of 2.4 nm and a total thickness of 14–15 mm. The membrane was placed between the receptor and donor compartments. Then, 0.5 g of hydrogel was placed on the membrane in the donor compartment. The acceptor compartment was filled with phosphate buffer (pH 7.4) and was maintained at 37 ± 0.5 °C and stirred by a magnetic bar at 200 ± 5 rpm. Next, 1 mL of the medium was withdrawn at intervals 15, 30, 45, 60, 90, 120, and 150 min (180, 210, 240, 270, 300 and 330 min in case of CS hydrogels). The volume of each sample was replaced by the same volume. Samples were analyzed for IBU content spectrophotometrically (UV Spectrophotometer (SHIMADZU^®^ UV–1900i) at λ_max_ = 264 nm.

Experimental results were expressed as mean ± SD (*n* = 7).

## Figures and Tables

**Table 1:** The values of the coefficient of determination (R^2^), flux (Jss), and permeation coefficient (K_p_) with standard deviation (SD) for the individual drug (IBU) release kinetic models of selected polysaccharide hydrogels.

Hydrogels	R^2^ (Zero-Order)	R^2^ (First-Order)	R^2^ (Higuchi)	R^2^ (Korsmeyer-Peppas)	R^2^ (Hixson-Crowell)	Jss (μg.cm^2^.h^−1^)	K_p_ × 10^3^ (cm.h^−1^) ± (SD)
4% MC	0.9520	0.9817	0.9878	0.9430	0.9331	48.103	1.896 ± 0.043
6% MC	0.9817	0.9901	0.9880	0.9719	0.9898	53.035	2.050 ± 0.070
2% HPMC	0.9636	0.9912	0.9661	0.9907	0.9841	53.693	2.118 ± 0.054
4% HEC	0.9507	0.9807	0.9907	0.9794	0.9730	55.710	2.213 ± 0.024
6% HEC	0.9687	0.9922	0.9977	0.9871	0.9863	48.899	1.8889 ± 0.026
6% NaCMC	0.9704	0.9768	0.9866	0.9863	0.9792	50.689	1.983 ± 0.023
8% CMC	0.9501	0.9853	0.9939	0.9802	0.9758	51.038	2.029 ± 0.008
10% CMC	0.9767	0.9937	0.9972	0.9832	0.9901	52.889	2.086 ± 0.012
2% TRG	0.9108	0.9694	0.9754	0.9455	0.9534	61.102	2.416 ± 0.014
4% TRG	0.9744	0.9967	0.9980	0.9901	0.9930	61.014	2.420 ± 0.016
6% TRG	0.9322	0.9703	0.9830	0.9769	0.9598	57.205	2.267 ± 0.014
8% CRG	0.9621	0.9912	0.9972	0.9899	0.9839	52.537	2.084 ± 0.011
10% CRG	0.9583	0.9847	0.9943	0.9926	0.9773	58.136	2.309 ± 0.012
4% XTG	0.9342	0.9775	0.9866	0.9815	0.9656	52.554	2.068 ± 0.006
6% XTG	0.9498	0.9842	0.9913	0.9714	0.9771	56.693	2.240 ± 0.014
2% CS HMW	0.9813	0.9800	0.9507	0.9290	0.9796	30.836	1.230 ± 0.002
3% CS HMW	0.9342	0.9298	0.8668	0.8434	0.7566	18.567	0.729 ± 0.017
2% CSc	0.9956	0.9931	0.9607	0.9740	0.9941	24.677	0.936 ± 0.011
3% CSc	0.9905	0.9908	0.9683	0.9903	0.9909	7.233	0.287 ± 0.002
2% CSs	0.9994	0.9976	0.9835	0.9405	0.9781	29.831	1.207 ± 0.048
3% CSs	0.9721	0.9721	0.9660	0.9689	0.9720	18.239	0.721 ± 0.004

## Data Availability

The data presented in this study are available on request from the corresponding author.

## Appendix A

**Table A1:** The building units of selected polysaccharides.

**Anionic Polysaccharides**	
Xanthan gum (XTG)	
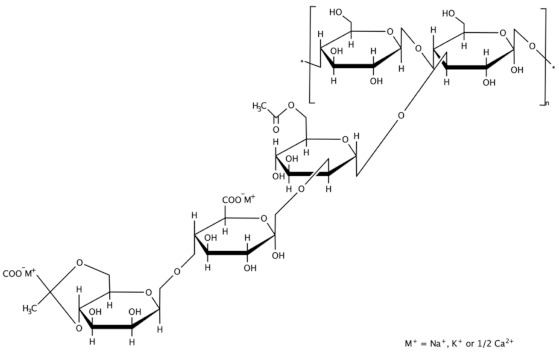
Tragacanth (TRG)	
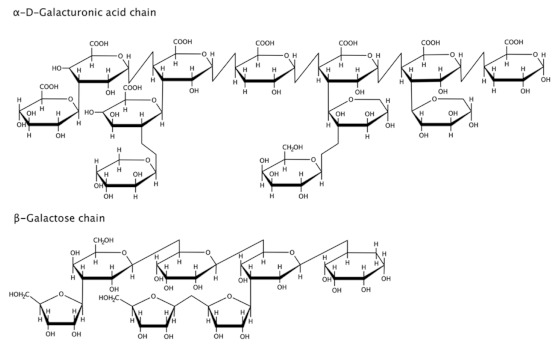
Carrageenan (CRG)	
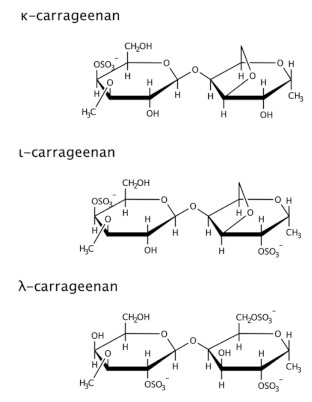
Carboxymethylcellulose (CMC)	
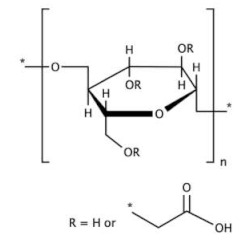
Sodium salt of carboxymethylcellulose (NaCMC)	
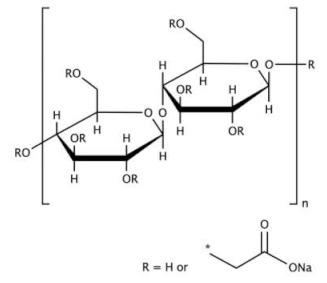
**Cationic Polysaccharides**	
Chitosan (CS)	
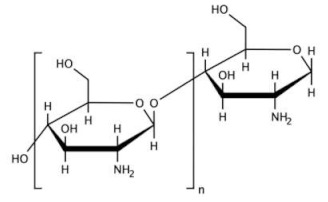
**Neutral Polysaccharides**	
Methylcellulose (MC)	
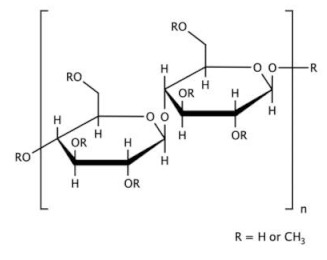
Hydroxypropylmethylcellulose (HPMC)	
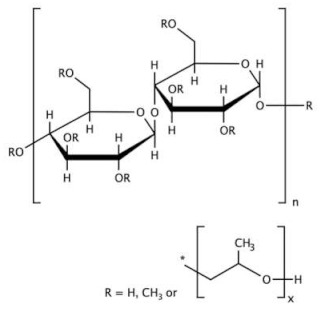
Hydroxyethylcellulose (HEC)	

## References

[1] Mikušová V., Mikuš P. (2021). Advances in Chitosan-Based Nanoparticles for Drug Delivery. Int. J. Mol. Sci..

[2] Vlachou M., Siamidi A. (2020). Biopolymers, liposomes, and nanofibers as modified peroral drug release formulants. Nanomaterials for Clinical Applications.

[3] Prasher P., Sharma M., Mehta M., Satija S., Aljabali A.A., Tambuwala M.M., Anand K., Sharma N., Dureja H., Jha N.K. (2021). Current-Status and Applications of Polysaccharides in Drug Delivery Systems. Colloid Interface Sci. Commun..

[4] D’Ayala G.G., Malinconico M., Laurienzo P. (2008). Marine Derived Polysaccharides for Biomedical Applications: Chemical Modification Approaches. Molecules.

[5] Kumarasamy D., Giri T.K., Maiti S., Jana S. (2019). 20—Biopolysaccharide-based hydrogel materials for drug delivery. Polysaccharide Carriers for Drug Delivery.

[6] Chopra H., Singh I., Kumar S., Bhattacharya T., Habibur R., Akter R., Kabir T. (2021). Current Drug Delivery A Comprehensive Review on Hydrogels. Curr. Drug Deliv..

[7] Zainal S.H., Mohd N.H., Suhaili N., Anuar F.H., Lazim A.M., Othaman R. (2021). Preparation of Cellulose-Based Hydrogel: A Review. J. Mater. Res. Technol..

[8] Shokri A.J., Adibki J.S., van de Ven T.A., Godbout L.B. (2013). Application of Cellulose and Cellulose Derivatives in Pharmaceutical Industries. Cellulose—Medical, Pharmaceutical and Electronic Applications.

[9] Yeo Y.H., Park W.H. (2021). Dual-Crosslinked, Self-Healing and Thermo-Responsive Methylcellulose/Chitosan Oligomer Copolymer Hydrogels. Carbohydr. Polym..

[10] Dharmalingam K., Anandalakshmi R. (2019). Fabrication, Characterization and Drug Loading Efficiency of Citric Acid Crosslinked NaCMC-HPMC Hydrogel Films for Wound Healing Drug Delivery Applications. Int. J. Biol. Macromol..

[11] Destruel P.-L., Zeng N., Seguin J., Douat S., Rosa F., Brignole-Baudouin F., Dufaÿ S., Dufaÿ-Wojcicki A., Maury M., Mignet N. (2020). Novel in Situ Gelling Ophthalmic Drug Delivery System Based on Gellan Gum and Hydroxyethylcellulose: Innovative Rheological Characterization, in Vitro and in Vivo Evidence of a Sustained Precorneal Retention Time. Int. J. Pharm..

[12] Ghorpade V.S., Yadav A.V., Dias R.J., Mali K.K., Pargaonkar S.S., Shinde P.V., Dhane N.S. (2018). Citric Acid Crosslinked Carboxymethylcellulose-Poly(Ethylene Glycol) Hydrogel Films for Delivery of Poorly Soluble Drugs. Int. J. Biol. Macromol..

[13] Dong Y., Wei Z., Xue C. (2021). Recent Advances in Carrageenan-Based Delivery Systems for Bioactive Ingredients: A Review. Trends Food Sci. Technol..

[14] Yegappan R., Selvaprithiviraj V., Amirthalingam S., Jayakumar R. (2018). Carrageenan Based Hydrogels for Drug Delivery, Tissue Engineering and Wound Healing. Carbohydr. Polym..

[15] Mahdavinia G.R., Karimi M.H., Soltaniniya M., Massoumi B. (2019). In Vitro Evaluation of Sustained Ciprofloxacin Release from κ-Carrageenan-Crosslinked Chitosan/Hydroxyapatite Hydrogel Nanocomposites. Int. J. Biol. Macromol..

[16] Nagaraja K., Rao K.M., Reddy G.V., Rao K.S.V.K. (2021). Tragacanth Gum-Based Multifunctional Hydrogels and Green Synthesis of Their Silver Nanocomposites for Drug Delivery and Inactivation of Multidrug Resistant Bacteria. Int. J. Biol. Macromol..

[17] Pathania D., Verma C., Negi P., Tyagi I., Asif M., Kumar N.S., Al-Ghurabi E.H., Agarwal S., Gupta V.K. (2018). Novel Nanohydrogel Based on Itaconic Acid Grafted Tragacanth Gum for Controlled Release of Ampicillin. Carbohydr. Polym..

[18] Peers S., Montembault A., Ladavière C. (2020). Chitosan Hydrogels for Sustained Drug Delivery. J. Control. Release.

[19] Mahanta A.K., Maiti P. (2019). Injectable Hydrogel through Hydrophobic Grafting on Chitosan for Controlled Drug Delivery. Acs Appl. Bio Mater..

[20] Patel A., Bell M., O’Connor C., Inchley A., Wibawa J., Lane M.E. (2013). Delivery of Ibuprofen to the Skin. Int. J. Pharm..

[21] Celebi D., Guy R.H., Edler K.J., Scott J.L. (2016). Ibuprofen Delivery into and through the Skin from Novel Oxidized Cellulose-Based Gels and Conventional Topical Formulations. Int. J. Pharm..

[22] Herkenne C., Naik A., Kalia Y.N., Hadgraft J., Guy R.H. (2007). Ibuprofen transport into and through skin from topical formulations: In vitro-in vivo comparison. J. Investig. Dermatol..

[23] Djekic L., Martinović M., Dobričić V., Čalija B., Medarević Đ., Primorac M. (2019). Comparison of the Effect of Bioadhesive Polymers on Stability and Drug Release Kinetics of Biocompatible Hydrogels for Topical Application of Ibuprofen. J. Pharm. Sci..

[24] Tsai T.S., Pillay V., Choonara Y.E., Du Toit L.C., Modi G., Naidoo D., Kumar P. (2011). A Polyvinyl Alcohol-Polyaniline Based Electro-Conductive Hydrogel for Controlled Stimuli-Actuable Release of Indomethacin. Polymers.

[25] Iglesias N., Galbis E., Valencia C., De-Paz M.V., Galbis J. (2018). Reversible pH-Sensitive Chitosan-Based Hydrogels. Influence of Dispersion Composition on Rheological Properties and Sustained Drug Delivery. Polymers.

[26] Špaglová M., Čuchorová M., Čierna M., Poništ S., Bauerová K. (2021). Microemulsions as Solubilizers and Penetration Enhancers for Minoxidil Release from Gels. Gels.

[27] Nakayama S., Ihara K., Senna M. (2009). Structure and Properties of Ibuprofen–Hydroxypropyl Methylcellulose Nanocomposite Gel. Powder Technol..

[28] Li R., Lyu Y., Luo S., Wang H., Zheng X., Li L., Ao N., Zha Z. (2021). Fabrication of a multi-level drug release platform with liposomes, chitooligosaccharides, phospholipids and injectable chitosan hydrogel to enhance anti-tumor effectiveness. Carbohydr. Polym..

[29] Mongkolkitikul S., Paradee N., Sirivat A. (2018). Electrically Controlled Release of Ibuprofen from Conductive Poly(3-Methoxydiphenylamine)/Crosslinked Pectin Hydrogel. Eur. J. Pharm. Sci..

[30] Jabeen S., Maswal M., Chat O.A., Rather G.M., Dar A.A. (2016). Rheological Behavior and Ibuprofen Delivery Applications of PH Responsive Composite Alginate Hydrogels. Colloids Surf. B Biointerfaces.

[31] Weng L., Rostamzadeh P., Jing S., Golzarian J. (2015). In vitro drug release from an ibuprofen-loaded biodegradable hydrogel. J. Vasc. Interv. Radiol..

[32] Münster L., Capáková Z., Fišera M., Kuřitka I., Vícha J. (2019). Biocompatible Dialdehyde Cellulose/Poly(Vinyl Alcohol) Hydrogels with Tunable Properties. Carbohydr. Polym..

[33] Dash S., Murthy P.N., Nath L., Chowdhury P. (2010). Kinetic Modeling on Drug Release from Controlled Drug Delivery Systems. Acta Pol. Pharm..

[34] Ghica M., Hîrjău M., Lupuleasa D., Dinu-Pîrvu C.E. (2016). Flow and Thixotropic Parameters for Rheological Characterization of Hydrogels. Molecules.

[35] Wróblewska M., Słyż J., Winnicka K. (2019). Rheological and textural properties of hydrogels, containing sulfur as a model drug, made using different polymers types. Polimery.

[36] Gadziński P., Osmałek T.Z., Froelich A., Wilmańska O., Nowak A., Tatarek A. (2022). Rheological and textural analysis as tools for investigation of drug-polymer and polymer–polymer interactions on the example of low-acyl gellan gum and mesalazine. J. Biomater. Appl..

[37] Zgoda M.M., Kołodziejska J. (2006). Effect of rheological parameters on pharmaceutical availability of ketoprofen from hydrogel products made on Carbopol base. Polim Med..

[38] Qandil A.M., Obaidat A.A., Mohammed Ali M.A., Al-Taani B.M., Tashtoush B.M., Al-Jbour N.D., Al Remawi M.M., Al-Sou’od K.A., Badwan A.A. (2009). Investigation of the Interactions in Complexes of LowMolecular Weight Chitosan with Ibuprofen. J. Sol. Chem..

[39] Puttipipatkhachorn S., Nunthanid J., Yamamoto K., Peck G.E. (2001). Drug physical state and drug–polymer interaction on drug release from chitosan matrix films. J. Control. Release.

[40] Bernkop-Schnürch A., Dünnhaupt S. (2012). Chitosan-based drug delivery systems. Eur. J. Pharm. Biopharm..

[41] Tajmir-Riahi H.A., Nafisi S., Sanyakamdhorn S., Agudelo D., Chanphai P., Jain K. (2014). Applications of Chitosan Nanoparticles in Drug Delivery. Drug Delivery System. Methods in Molecular Biology (Methods and Protocols).

[42] Su C., Daqing W., Yu Z. (2011). Mechanical and corrosion resistance of hydrophilic sphene/titania composite coatings on titanium and deposition and release of cefazolin sodium/chitosan films. Appl. Surf. Sci..

[43] Veeramachineni A.K., Sathasivam T., Paramasivam R., Muniyandy S., Pushpamalar J. (2019). Synthesis and Characterization of a Novel pH-Sensitive Aluminum Crosslinked Carboxymethyl Tragacanth Beads for Extended and Enteric Drug Delivery. J. Polym. Environ..

[44] Djekic L., Martinović M., Ćirić A., Fraj J. (2020). Composite Chitosan Hydrogels as Advanced Wound Dressings with Sustained Ibuprofen Release and Suitable Application Characteristics. Pharm. Dev. Technol..

